# ProFold: Protein Fold Classification with Additional Structural Features and a Novel Ensemble Classifier

**DOI:** 10.1155/2016/6802832

**Published:** 2016-08-28

**Authors:** Daozheng Chen, Xiaoyu Tian, Bo Zhou, Jun Gao

**Affiliations:** ^1^College of Information Engineering, Shanghai Maritime University, Shanghai 201306, China; ^2^Shanghai University of Medicine & Health Sciences, Shanghai 201318, China

## Abstract

Protein fold classification plays an important role in both protein functional analysis and drug design. The number of proteins in PDB is very large, but only a very small part is categorized and stored in the SCOPe database. Therefore, it is necessary to develop an efficient method for protein fold classification. In recent years, a variety of classification methods have been used in many protein fold classification studies. In this study, we propose a novel classification method called proFold. We import protein tertiary structure in the period of feature extraction and employ a novel ensemble strategy in the period of classifier training. Compared with existing similar ensemble classifiers using the same widely used dataset (DD-dataset), proFold achieves 76.2% overall accuracy. Another two commonly used datasets, EDD-dataset and TG-dataset, are also tested, of which the accuracies are 93.2% and 94.3%, higher than the existing methods. ProFold is available to the public as a web-server.

## 1. Introduction

Protein fold classification is a crucial problem in structural bioinformatics. Protein folding information is helpful in identifying the tertiary structure and functional information of a protein [1]. In recent years, many protein fold classification studies have been performed. The methods proposed by researchers can be roughly divided into two categories: one is template-based method [2–7], and the other is taxonomy-based method [8–15]. Recently, taxonomy-based methods have attracted more attention due to their relatively excellent performance.

The taxonomy-based method was proposed by Dubchak et al. [8, 9] in 1995 for the first time. Many taxonomy-based methods classify a query protein to a known folding type. This nonmanual label method contributes to the growth of the quantity of protein in Structural Classification of Proteins (SCOP) [16] and could narrow the gap between the number of proteins in SCOP and Protein Data Bank (PDB). In this paper, the taxonomy-based method is equivalent to the classification problem in machine learning. There are two significant problems in classification tasks: one is feature extraction, and the other is machine learning method.

In terms of feature extraction, most of the researchers extract multidimensional numerical feature vectors from amino acid sequences. In 1995, Dubchak et al. [8, 9] extracted global description of amino acid sequence for the first time. Since then, in order to improve the accuracy of classification, some researchers have put forward other feature extraction methods, such as pseudoamino acid composition [12, 17], pairwise frequency information [18], Position Specific Scoring Matrix (PSSM) [17], structural properties of amino acid residues and amino acid residue pairs [19], and hidden Markov model structural alphabet [20, 21]. Except for extracting features from amino acid sequence directly, some features are extracted from evolution information combining the functional domain and the sequential evolution information [22] and predicted secondary structure [14, 23, 24]. Although the classification accuracy can be improved after combining these features together [20, 25], it is still not good enough.

For protein fold classification, many classifiers have been used, such as neutral network (NNs) [8, 13], SVMs [10, 13, 18–21, 24, 26–33],* k*-nearest neighbors (*k*-NN) [12], probabilistic multiclass multikernel classifier [25], random forest [23, 34–37], rotation forest [38], and a variety of ensemble classifiers [11, 12, 14, 18, 22, 39–41].

Up to 28th April, 2016, PDB had 109850 protein structures (http://www.rcsb.org/pdb/home/home.do). However, Structural Classification of Proteins- extended (SCOPe) [42] only had 77439 PDB entries (http://scop.berkeley.edu/statistics/ver=2.06). Therefore, there still exists a great number of protein structures which do not have their structure classification labels in the SCOPe database. What is more, most protein structures in SCOPe are classified manually, so it requires a lot of manual labor. In this study, we start from the PDB file 3D structure studying the protein fold classification. In terms of feature extraction, we use a new feature extraction method, combining the existing methods of the global description of amino acid sequence [13], PSSM [43], and protein functional information [22] proposed by other researchers. The new feature extraction method extracts eight types of secondary structure states from PDB files by the Definition of Secondary Structure in Proteins (DSSP) software [44]. In terms of machine learning classifiers, we propose a novel ensemble strategy. With the new added feature extracted from DSSP and the novel ensemble strategy we propose, our method can achieve 1–3% higher accuracy than similar methods.

As demonstrated by a series of recent publications [45–55] in compliance with Chou's 5-step rule [56], to establish a really useful machine learning classifier for a biological system, we should follow the following five guidelines: (a) benchmark dataset construction or selection for training and testing the model; (b) extract features from the biological sequence samples with effective methods that can truly reflect their intrinsic correlation with the target to be predicted; (c) introduce or develop a powerful algorithm (or engine) to operate the classifier; (d) properly perform cross-validation tests and test on independent dataset to objectively evaluate the anticipated accuracy of the classifier; (e) establish a user-friendly web-server (http://binfo.shmtu.edu.cn/profold/) for the classifier that is accessible to the public. In the following, we are to describe how to deal with these steps one-by-one.

## 2. Materials and Methods

### 2.1. Data Sets

In this study, three benchmark datasets are used, respectively: (1) Ding and Dubchak (DD) [13], (2) Taguchi and Gromiha (TG) [58], and (3) Extended DD (EDD) [10]. DD-dataset was proposed by Ding and Dubchak in 2001 and modified by Shen and Chou in 2006 [12]. Since then, DD-dataset has been used in many protein fold classification studies [11, 18, 20–24, 26, 32–36, 38, 40, 57, 59]. There are 311 protein sequences in the training set and 386 protein sequences in the testing set with no two proteins having more than 35% of sequence identity. The protein sequences in DD-dataset were selected from 27 SCOP [35] folds comprehensively, which belong to different structural classes containing *α*, *β*, *α*/*β*, and *α* + *β*.

TG-dataset contains 30 SCOP folds and 1612 protein sequences with no two protein having more than 25% sequence identity.

EDD-dataset contains 27 SCOP folds, like DD-dataset. There are 3418 protein sequences with no protein having more than 40% sequence identity.

These three datasets can be downloaded directly from our website (http://binfo.shmtu.edu.cn/profold/benchmark.html).

### 2.2. Feature Extraction Method

With the rapid growth of biological sequences in the postgenomic age, one of the most important but also most difficult problems in computational biology is how to represent a biological sequence with a discrete model or a vector. Therefore Chou's PseAAC [60–62] was proposed. Encouraged by the successes of using PseAAC to deal with protein/peptide sequences, three web-servers [63–65] were developed for generating various feature vectors for DNA/RNA sequences. Particularly, recently a powerful web-server called Pse-in-One [66] has been established that can be used to generate any desired feature vectors for protein/peptide and DNA/RNA sequences according to the need of users' studies. Inspired by this, in this study, we extract four feature groups, including the DSSP feature, the amino acid composition and physicochemical properties (AAsCPP) feature, the PSSM feature, and the functional domain (FunD) composition feature. These feature extraction methods will be described as follows.

#### 2.2.1. Definition of Secondary Structure in Proteins

The DSSP program was designed by Kabsch and Sander [44] and used to standardize protein secondary structure. The DSSP program works by calculating the most likely protein secondary structure given by the protein 3-dimensional structure. The specific principle of the DSSP program is calculating the H-bond energy between every two atoms by the atomic position in a PDB file, and then the most likely class of secondary structure for each residue can be determined by the best two H-bonds of each atom.

The DSSP feature extraction process is as follows. Firstly, DSSP entries are calculated from PDB entries by DSSP program. Secondly, the corresponding DSSP sequences from DSSP entries are obtained. DSSP sequence contains eight states (T, S, G, H, I, B, E, —), which can be divided into four groups, as shown in Table 1. Finally, according to the eight states and four groups, a 40D feature vector can be extracted from a DSSP sequence. The detail of the description and dimension of the features are shown in Table 2.

#### 2.2.2. Amino Acids Composition and Physicochemical Properties

As effective features to describe a protein, the amino acid composition and physiochemical properties have reached good predict result, respectively [13, 34, 35]. Ding and Dubchak [13] tried to integrate the features for the first time and achieved a good result. Later, many other researchers proposed other feature integration methods. In 2013, Lin et al. [41] used a 188D feature vector combining amino acid composition and physiochemical properties. The 188D feature extraction method is also used in this paper.

The eight physiochemical properties of amino acids are hydrophobicity, van der Waals volume, polarity, polarizability, charge, surface tension, surface tension, and solvent accessibility. Different kinds of amino acids have different physiochemical properties so that they can be divided into three groups [13, 41], as shown in Table 3.

The percentage composition of the 20 amino acids in the query protein forms a 20D feature vector. The group composition of amino acids (3D), the pairwise frequency between every two groups (3D), and the distribution pattern of constituents (where the first, 25%, 50%, 75%, and 100% of a given constituent are contained) (5 × 3D) from each physiochemical property are extracted. Therefore, we can get a 168D feature vector from a protein sequence according to the eight physiochemical properties. Adding up the 20D amino acid composition feature and the 168D physiochemical feature, we can get a 188D feature vector altogether. The name and the dimensions of the features are listed in Table 4.

#### 2.2.3. Position Specific Scoring Matrix

PSSM is a relatively common feature. In addition to protein fold type classification research area, there are some studies on protein structural class prediction [67, 68] which used this feature. PSSM is derived from PSI-BLAST (Position Specific Iterative Basic Local Alignment Search Tool) [43] by taking the multiple sequence alignment of sequences in nonredundant protein sequence database (nrdb90) [69]. The iteration number is 3 and the cutoff *E*-value is 0.001. Two *L* × 20 matrices can be obtained by PSI-BLAST, in which *L* represents the length of the query amino acid sequence, and 20 represents the 20 amino acids. One of the two matrices contains conservation scores of a given amino acid at a given position in sequence, and the other provides probability of occurrence of a given amino acid at a given position in the sequence. The PSSM feature is extracted from the former matrix. Suppose that the parameter in the matrix is *S*_*ij*_  (*i* = 1,2,…, *L*;  *j* = 1,2,…, 20). Then the feature can be calculated by (1). That is to calculate the average value of each column in the matrix and form a 20D feature vector.

(1) $$\begin{array}{c} P_{\text{pssm}}={\left[\;\frac{\sum_{i=1}^{L}S_{i1}}{L},\frac{\sum_{i=1}^{L}S_{i1}}{L},\ldots ,\frac{\sum_{i=1}^{L}S_{i20}}{L}\;\right]}^{T}. \end{array}$$

#### 2.2.4. Functional Domain Composition

Proteins always contain some modules or domains, which involve different evolution resources and functions. Therefore, we can extract features in some FunD databases. There are some different FunD databases: SMART [70], Pfam [71], COG [72], KOG [72], and CDD [73]. In 2009, Shen and Chou [22] considered CDD as a relatively more complete functional domain database, and they used CDD to extract features. In this study, we used CDD (version 2.11), which co ntains 17402 common protein domains and families. Taking each of protein domains as a vector-base, we can extract a 17402D feature vector. Specific process is as follows. Firstly, use RPS-BLAST program [74] to compare the protein sequence with each of the 17402 domain sequences. Secondly, if the significance threshold value (expect value) is no more than 0.001, this component of the protein in the 17402D feature vector is assigned 1; otherwise, it is assigned 0. In this way, we can extract a 17402D feature vector, and each component of the feature can be either 1 or 0.

### 2.3. The Proposed Ensemble Classifier

**Figure 1:**
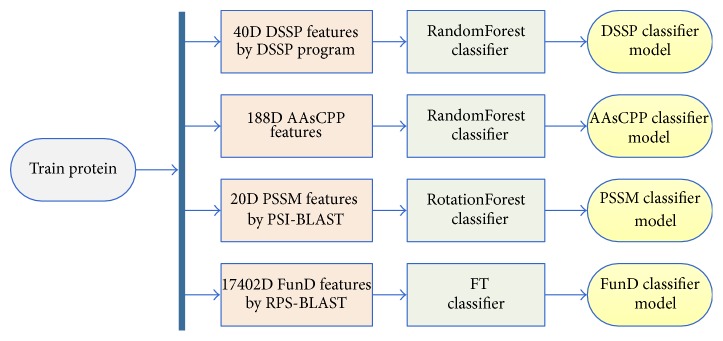
The training process of the four feature groups through the corresponding classifier.

**Figure 2:**
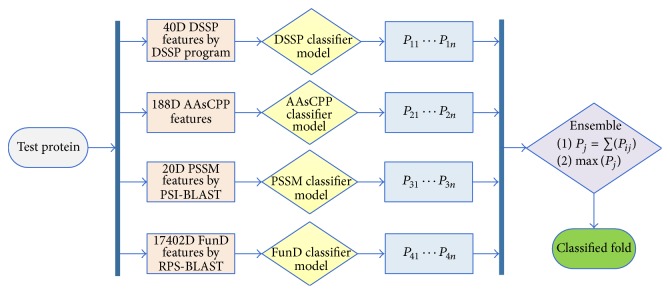
The ensemble process of calculating the test data through the models.

In this study, we propose a novel ensemble strategy which includes 5 individual steps. Step 1: 10 widely used machine learning classifiers, LMT [75], RandomForest [34], LibSVM [76], SimpleLogistic [75], RotationForest [38], SMO [77], NaiveBayes [78], RandomTree [79], FT [80], and SimpleCart [81], are selected, and a 5-fold cross validation is implemented on the DD-dataset. Step 2: the classifier with the highest accuracy in each feature group is chosen. Step 3: corresponding models by training each feature group with the chosen classifier are selected. The four models are DSSP classification model, AAsCPP classification model, PSSM classification model, and FunD classification model. Detailed process is shown in Figure 1. Step 4: features from the test dataset are extracted and the classification result *P*_*ij*_ by calculating the corresponding models is obtained, *i* represents a kind of classification model ranging from 1 to 4, and *j* represents a kind of fold index, ranging from 1 to the total number of the fold classes (e.g., the value of *j* ranges from 1 to 27 on DD-dataset). Step 5: the average of the probabilities of the four models in each fold class is calculated. The fold class with the highest probability will be chosen as the classification result. Detailed process is shown in Figure 2.

The machine learning tool we used is WEKA (Waikato Environment for Knowledge Analysis) [56], a collection of machine learning classifiers for data mining tasks based on Java.

### 2.4. Measurement

In this study, the standard *Q* percentage accuracy is used to test the effect of the proposed classification method, which helped us to compare our result with other researchers' results [12, 13, 34]. The definition of the standard *Q* percentage accuracy is described in

(2) N=n1+n2+⋯+ni+⋯+nk,C=c1+c2+⋯+ci+⋯+ck,Q=CN,

where *n*_*i*_ represents the number of the proteins which belong to class *i*, *c*_*i*_ represents the correct number in *n*_*i*_ test data, *c*_*i*_/*n*_*i*_ represents the classification accuracy of class *i*, *k* represents the total number of classes, *N* represents the total number of tests, *C* represents the total number of the correct classified data, and *Q* represents the classification accuracy.

## 3. Results and Discussion

### 3.1. Performance of ProFold

In order to test the performance of proFold, we first select the widely used DD-dataset for evaluation. The overall accuracy is 76.2%. Comparison with existing ensemble learning methods on DD-dataset is shown in Table 5. From Table 5, we can see that the accuracy of the other methods are under 75%, and the accuracy of our method is 3% higher than PFPA (2015) [40], which is the best one in the other methods.

In order to further evaluate the performance of proFold, we also select another two large scale datasets: EDD-dataset and TG-dataset. Training and testing dataset are not clearly distinguished in the two datasets, so a *k*-fold cross validation is implemented on them.

We calculated the classification accuracy of EDD-dataset by 10-fold cross validation for 10 times and compared the result with other methods. The results are shown in Table 6. We can see from the table that only the accuracies of Paliwal et al. and Lyons et al. are more than 90%, which are lower than that of proFold. The result showed that the advantage of proFold is obvious when larger scale datasets are used for validation.

Regarding TG-dataset, we also took experiments by 10-fold cross validation for 10 times and compared the results with other methods. The results are shown in Table 7. We can see from the table that HMMFold (2015) method achieved the highest accuracy, which is 93.8%. The accuracy of our method is 94.3%, which is higher than HMMFold. TG-dataset has threefold classes more than DD-dataset and its scale is twice larger than DD-dataset. The result showed that the advantage of proFold is obvious when the dataset with more fold classes is tested.

### 3.2. Performance of the Proposed Ensemble Classifier

In the field of protein fold classification, many researchers used ensemble learning methods [11, 18, 22, 23, 34–36, 38, 46, 51, 54, 79, 82–89]. The specific process of those ensemble strategies is as follows. (1) Integrate all features. (2) Select several basic classifiers for training. (3) Propose an ensemble classifier according to the classification result probability of each basic classifier. In this study, we find that the redundancies of the features will influence the performance of those methods. Therefore, we propose a novel ensemble strategy.

We took experiments on DD-dataset. Firstly, extract four feature groups which have been tested in 10 basic classifiers by cross validation. The detailed information of the test results is listed in Table 8. We can see from Table 8 that the best classifier is RandomForest using the DSSP feature group and AAsCPP feature group. The best classifiers are RotaionForest and FT when PSSM and FunD features are implemented, respectively. Secondly, train the four feature groups with corresponding basic classifiers and get four models. Finally, test the models on DD-dataset. The overall accuracy is 76.2%. Our method improves the accuracy effectively compared with other existing ensemble learning methods.

In order to compare our ensemble strategy with the traditional ensemble strategy, we took experiments on the four feature groups with traditional ensemble strategy. (1) Integrate the four feature groups. (2) Train the models with RandomForest, RotationForest, and FT respectively. (3) Test the models on DD-dataset, EDD-dataset, and TG-dataset. The classification accuracy of our ensemble strategy has increased by 3% to 4%, as shown in Table 9. The result showed that our ensemble strategy has a better classification performance.

### 3.3. Accuracy Improvements with the DSSP Feature

In order to evaluate the influence on importing the DSSP feature, we calculated the classification accuracy of each fold class with and without the DSSP feature, respectively, using the DD-dataset. The accuracies are shown in Table 10. From the table, we can see that the accuracies of some fold classes, such as Fold number 2, number 4, number 6, number 12, number 23, and number 26, have increased obviously after importing the DSSP feature. The overall accuracy has increased from 71.3% to 76.2%. For example, the protein chain 1FAPB in DD-dataset was incorrectly classified into Fold number 5 before importing the DSSP feature, and it was reclassified into Fold number 4 correctly after importing the DSSP feature. The results showed that the DSSP feature has a significant effect on protein structure classification.

As we know that PDB files contain protein 3D structure information, we started from the PDB file of the protein in this study. The DSSP feature is extracted from the 3D structure in PDB and the 3D structure of a protein is more stable. Thus it explains why the DSSP feature has a significant effect on the protein structure classification.

## 4. Conclusion

In this study, we proposed a novel method called proFold. ProFold is an ensemble classifier combining the protein structural and functional information. In terms of feature extraction, we imported the DSSP feature into protein fold classification for the first time. Experiments showed that the classification accuracy will increase by about 5% using the DD-dataset by importing the DSSP feature. In terms of classification method, we proposed a novel ensemble classifier and improved the classification accuracy with this method. The classification accuracies of proFold on DD-, EDD-, and TG-dataset are 76.2%, 93.2%, and 94.3%, respectively, which are higher than the existing similar methods. The results showed that proFold is a relatively better classifier.

## Figures and Tables

**Table 1:** The eight states of DSSP feature in four groups.

Eight-state SS	Code	Description	Four groups
3_10_ helix (G)	G	Helix-3	First
Alpha-helix (H)	H	Alpha helix	
pi-helix (I)	I	Helix-5	

Beta-strand (E)	E	Strand	Second
Beta-bridge (B)	B	Beta bridge	

Beta-turn (T)	T	Turn	Third
High curvature loop (S)	S	Bend	

Irregular (L)	—	Empty, no secondary structure assigned	Fourth

**Table 2:** The description and dimension of the DSSP feature.

Features description	Dimension
State composition	8
Group composition	4
Number of continuous states	8
Number of continuous groups	4
Number of continuous state compositions	8
Number of continuous group compositions	4
Alternate frequency between groups	4

**Table 3:** The 20 amino acids divided into 3 groups according to their physiochemical properties.

Physicochemical property	The 1st group	The 2nd group	The 3rd group
Hydrophobicity	RKEDQN	GASTPHY	CVLIMFW
Van der Waals volume	GASCTPD	NVEQIL	MHKFRYW
Polarity	LIFWCMVF	PATGS	HQRKNED
Polarizability	GASDT	CPNVEQIL	KMHFRYW
Charge	KR	ANCQGHILMFPSTWYV	DE
Surface tension	GQDNAHR	KTSEC	ILMFPWYV
Secondary structure	EALMQKRH	VIYCWFT	GNPSD
Solvent accessibility	ALFCGIVW	RKQEND	MPSTHY

**Table 4:** The name and the dimension of the amino acids composition and physiochemical features.

Feature name	Dimension
Amino acids composition	20
Hydrophobicity	21
Van der Waals volume	21
Polarity	21
Polarizability	21
Charge	21
Surface tension	21
Secondary structure	21
Solvent accessibility	21

**Table 5:** Comparison with existing ensemble learning methods on DD-dataset.

Methods	References	Overall accuracy (%)
PFP-Pred	[12]	62.1
GAOEC	[11]	64.7
ThePFP-FunDSeqE	[22]	70.5
Dehzangi et al.	[34]	62.7
Dehzangi et al.	[38]	62.4
MarFold	[18]	71.7
PFP-RFSM	[35]	73.7
Feng and Hu	[36]	70.2
Feng et al.	[23]	70.8
PFPA	[40]	73.6
*proFold (the proposed method)*	*This paper*	*76.2*

**Table 6:** Comparison with the different methods on EDD-dataset by 10-fold cross validation.

Methods	References	Overall accuracy (%)
Paliwal et al.	[29]	90.6
Paliwal et al.	[30]	86.2
Dehzangi et al.	[31]	88.2
HMMFold	[32]	86.0
Saini et al.	[33]	89.9
Lyons et al.	[21]	92.9
*proFold (the proposed method)*	*This paper*	*93.2*

**Table 7:** Comparison with the different methods on TG-dataset by 10-fold cross validation.

Methods	References	Overall accuracy (%)
Paliwal et al.	[29]	77.0
Paliwal et al.	[30]	73.3
Dehzangi et al.	[31]	73.8
HMMFold	[32]	93.8
Saini et al.	[33]	74.5
NiRecor	[57]	84.6
Lyons et al.	[21]	85.6
*proFold (the proposed method)*	*This paper*	*94.3*

**Table 8:** The accuracy of 5-fold cross validation on the features extracted from DD-dataset using 10 basic classifiers.

Feature groups	Basic classifiers	Fivefold CV accuracy (%)
DSSP	LMT	43.0
	RandomForest^*∗*^	51.3
	LibSVM	46.4
	SimpleLogistic	43.0
	RotationForest	49.7
	SMO	36.4
	NaiveBayes	43.4
	RandomTree	32.8
	FT	42.4
	SimpleCart	37.7

AAsCPC	LMT	32.5
	RandomForest^*∗*^	35.4
	LibSVM	34.4
	SimpleLogistic	32.5
	RotationForest	27.7
	SMO	34.4
	NaiveBayes	28.3
	RandomTree	11.6
	FT	34.4
	SimpleCart	20.6

PSSM	LMT	56.3
	RandomForest	53.7
	LibSVM	57.2
	SimpleLogistic	55.9
	RotationForest^*∗*^	56.1
	SMO	30.2
	NaiveBayes	42.4
	RandomTree	29.6
	FT	49.5
	SimpleCart	33.4

FunD	LMT	42.1
	RandomForest	43.1
	LibSVM	21.2
	SimpleLogistic	43.1
	RotationForest	41.8
	SMO	38.9
	NaiveBayes	38.3
	RandomTree	39.9
	FT^*∗*^	44.1
	SimpleCart	34.7

^*∗*^The basic classifier of each feature group with the highest accuracy.

**Table 9:** Comparison with the different ensemble strategies on three datasets.

Datasets	The accuracy of traditional ensemble strategy (%)	The accuracy of this paper ensemble strategy (%)
DD	72.5	76.2
EDD	89.9	93.2
TG	91.7	94.3

**Table 10:** The accuracy of each fold class with and without the DSSP feature.

Fold number	The accuracy without the DSSP feature	The accuracy with the DSSP feature
1	100.0	100.0
2^*∗*^	88.9	100.0
3^*∗*^	55.0	60.0
4^*∗*^	62.5	87.5
5	88.9	88.9
6^*∗*^	66.7	77.8
7^*∗*^	77.3	84.1
8	66.7	66.7
9	92.3	92.3
10	66.7	66.7
11	50.0	50.0
12^*∗*^	47.4	68.4
13	100.0	100.0
14	50.0	50.0
15	100.0	100.0
16^*∗*^	91.7	93.8
17^*∗*^	83.3	91.7
18^*∗*^	38.5	46.2
19	85.2	85.2
20	50.0	50.0
21	87.5	87.5
22	58.3	58.3
23^*∗*^	57.1	71.4
24	100.0	100.0
25	25.0	25.0
26^*∗*^	44.4	59.3
27^*∗*^	92.6	96.3

Overall	71.3	76.2

^*∗*^The fold class of which the accuracy has increased significantly after importing the DSSP feature.
